# Inhibition of RhoA and Cdc42 by miR-133a Modulates Retinoic Acid Signalling during Early Development of Posterior Cardiac Tube Segment

**DOI:** 10.3390/ijms23084179

**Published:** 2022-04-10

**Authors:** Carlos Garcia-Padilla, Virginio Garcia-Lopez, Amelia Aranega, Diego Franco, Virginio Garcia-Martinez, Carmen Lopez-Sanchez

**Affiliations:** 1 Department of Human Anatomy and Embryology, Faculty of Medicine, Institute of Molecular Pathology Biomarkers, University of Extremadura, 06006 Badajoz, Spain; carlosgp@unex.es (C.G.-P.); garcialopez@unex.es (V.G.-L.); virginio@unex.es (V.G.-M.); 2 Department of Experimental Biology, University of Jaen, 23071 Jaen, Spain; aaranega@ujaen.es (A.A.); dfranco@ujaen.es (D.F.); 3 Fundación Medina, 18016 Granada, Spain

**Keywords:** miR-133a, retinoic acid signalling, RhoA, Cdc42, Raldh2, cardiac development, atrial differentiation

## Abstract

It is well known that multiple microRNAs play crucial roles in cardiovascular development, including miR-133a. Additionally, retinoic acid regulates atrial marker expression. In order to analyse the role of miR-133a as a modulator of retinoic acid signalling during the posterior segment of heart tube formation, we performed functional experiments with miR-133a and retinoic acid by means of microinjections into the posterior cardiac precursors of both primitive endocardial tubes in chick embryos. Subsequently, we subjected embryos to whole mount in situ hybridisation, immunohistochemistry and qPCR analysis. Our results demonstrate that miR-133a represses RhoA and Cdc42, as well as Raldh2/Aldh1a2, and the specific atrial markers *Tbx5* and *AMHC1*, which play a key role during differentiation. Furthermore, we observed that miR-133a upregulates p21 and downregulates cyclin A by repressing RhoA and Cdc42, respectively, thus functioning as a cell proliferation inhibitor. Additionally, retinoic acid represses miR-133a, while it increases Raldh2, *Tbx5* and *AMHC1*. Given that RhoA and Cdc42 are involved in Raldh2 expression and that they are modulated by miR-133a, which is influenced by retinoic acid signalling, our results suggest the presence of a negative feedback mechanism between miR-133a and retinoic acid during early development of the posterior cardiac tube segment. Despite additional unexplored factors being possible contributors to this negative feedback mechanism, miR-133a might also be considered as a potential therapeutic tool for the diagnosis, therapy and prognosis of cardiac diseases.

## 1. Introduction

Cardiac development is a complex morphogenetic process originating at the cardiac precursor cell level, located in the epiblast and primitive streak during early chick gastrulation stages. Precardiac cells invaginate through the primitive streak and migrate anterolaterally to both sides of the embryo, constituting the precardiac mesoderm between the ectoderm and the adjacent inductive endoderm [1,2,3,4,5,6,7]. In later stages, these fields form both primitive endocardial tubes, then fusing in the midline into a single heart tube and developing into the cardiac looping. Its anterior and posterior segments differentiate simultaneously, giving rise to the common ventricle and outflow tract, and the common *atrium* and inflow tract, respectively [8,9,10,11,12].

These morphogenetic processes are monitored by different molecular signalling pathways, including retinoic acid (RA), which has been demonstrated as necessary to define the anterior/posterior polarity of the heart and to form and differentiate the *atrium* and inflow tract [13,14,15,16,17,18]. RA synthesis is a critical process controlled largely by retinaldehyde dehydrogenase 2 (Raldh2/Aldh1a2) during heart development [19,20,21,22]. Interestingly, in the lateral mesoderm, Raldh2 conveys RA signalling to cardiac precursors, which is subsequently required to determine cardiac anteroposterior specification [23]. During initial posterior myocardium differentiation takes place Raldh2 synthesis, coinciding with activation of the atrial-specific myosin heavy chain (*AMHC1*) gene, an RA responsive marker of the posterior heart segment [24]. Furthermore, Raldh2-/- mutant hearts display cardiomyocyte hypoplasia defects with poor development of the *atrium* and *sinus venosus* due to lack of RA function [25,26]. This defect is correlated with abnormal expression levels and spatial distribution of the T-box gene *Tbx5*, a marker of the prospective atrial and *sinus venosus* regions [27,28]. In this sense, Raldh2 is already widely considered a pivotal protein involved in retinol active form biosynthesis [29,30,31]. In a similar line of research, it has been found that the Rho GTPase family (RhoA, Cdc42 and Rac1) is needed for Raldh2 to be expressed during cardiac development. In this sense, transgenic hearts characterised by an increased cardiac expression of Rho GDI (Rho GDP Dissociation Inhibitors), which is an inhibitor of Rho GTPase family activity, show diminished Raldh2 expression. Consequently, inappropriate early cardiogenesis, characterised by alteration of cardiac looping, loss of boundaries between cardiac chambers and cardiomyocyte hypoplasia, induces embryonic lethality [32]. Moreover, Rho GTPase is necessary for cardiomyocyte proliferation: (i) Cdc42 (cell division cycle 42) has been shown to induce cyclin A expression [33] and (ii) RhoA induces cyclin A expression by downregulating p21, a repressor of cyclin A expression [34,35].

As part of the above molecular network, different microRNAs are involved in several cardiogenic phases [36,37,38,39,40]. In particular, miR-133a contributes to differentiation, proliferation, survival and electrical conduction of cardiac cells [41,42,43,44,45]. In this sense, in humans and mice, RhoA and Cdc42 have been identified as specific target genes downregulated by miR-133a [46]. Thus, miR-133a downregulation causes cardiac hypertrophy, indicating that this microRNA functions as a negative regulator of cardiomyocyte proliferation [46]. Additionally, previous reports pointed out several microRNAs as significant upstream and downstream modulators of RA signalling, playing a pivotal role in RA-dependent biological processes [31,47,48].

In this work, by means of gain- and loss-of-function experiments, we analyse the interactive roles between miR-133a and RA signalling, involving RhoA and Cdc42, Rho GTPases Raldh2 inductors, in cardiac tube differentiation. We also analysed the role of miR-133a as cardiomyocyte proliferation modulator through p21 and cyclin A expressions. Our results reveal a negative feedback mechanism between miR-133a and RA during early development of the posterior cardiac tube segment.

## 2. Results

In this research study, we obtained several results that altogether provide original data about RA pathway signalling modulatory factors during differentiation of the posterior cardiac tube segment. Our findings revealed that miR-133a plays a crucial role in RA signalling during the early genetic programme of the sino-atrial region. A novel contribution of this work also includes the analysis of miR-133a expression pattern during early cardiac looping. Moreover, in this process, we have identified the RhoA and Cdc42 distribution and Rho GTPases previously reported [32] as modulators of Raldh2, which is necessary for RA synthesis, during heart development. Through gain-of-function experiments, we observed that miR-133a downregulates RhoA and Cdc42 directly, decreasing Raldh2, and that the expression of specific atrial gene *Tbx5* and *AMHC1* are also diminished. Additionally, we found that miR-133a modulates p21 and cyclin A, previously reported in cell proliferation processes [34,35].

In this work, we further analysed the effect of RA administration on miR-133a expression, as well as on Raldh2, and atrial specific genes, *Tbx5* and *AMHC1*, expression.

### 2.1. miR-133a Represses RA Synthesis by RhoA and Cdc42 Modulation

**Figure 1:**
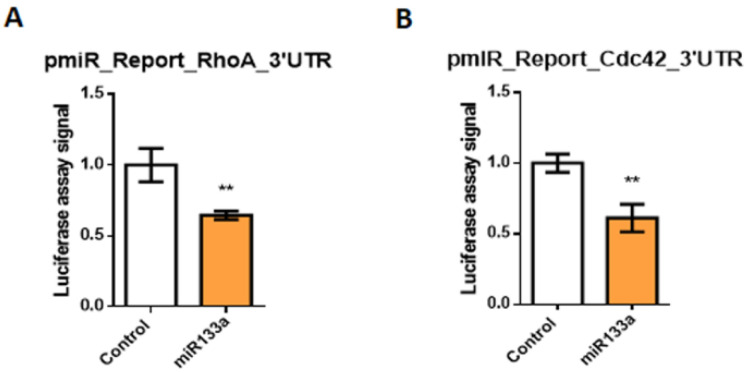
Representative data of RhoA (**A**) and Cdc42 (**B**) 3′UTR luciferase assays after premiR-133a overexpression in 3T3 fibroblasts. Luciferase activity was compared to non-transfected controls. Each luciferase assay was carried out in triplicate. Student’s *t*-test: ** *p* < 0.01.

In order to understand miR-133a modulating mechanisms involved in RA signalling during posterior cardiac tube differentiation, we analysed the miR-133a targets. Bioinformatics analyses through Target-Scan software show that RhoA and Cdc42 3′UTRs are putative targets of miR-133a (Supplementary Figure S1), suggesting that they might exert post-transcriptional regulation of both Rho GTPases. Our luciferase assays show that luciferase signals from plasmids harbouring 3′UTR of each gene are reduced with respect to control, demonstrating that miR-133a is able to recognise and directly bind to RhoA and Cdc42 3′UTR, triggering their mRNA degradation (Figure 1).

**Figure 2:**
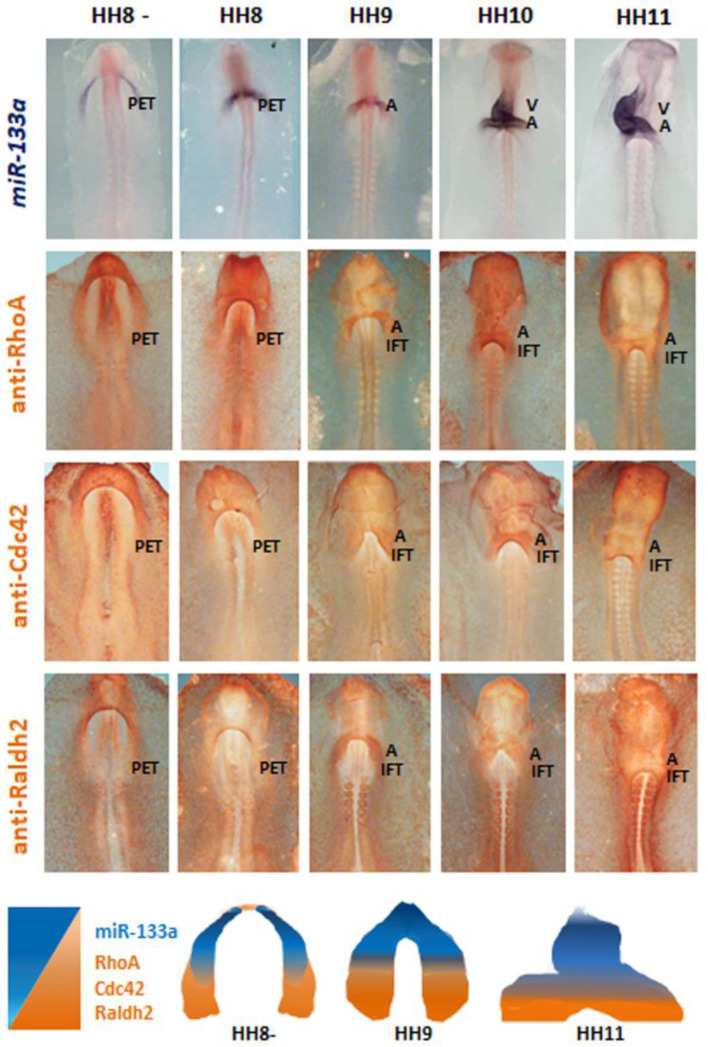
Whole-mount ISH for miR-133a and whole mount IMH for RhoA, Cdc42 and Raldh2 during early chick cardiac development, from HH8–HH11 stages, in control embryos. Note miR-133a expression pattern at the level of the anterior region of the primitive endocardial tube (PET), being observable in the *atrium* (A) and ventricle (V) at later stages. Note the location of RhoA, Cdc42 and Raldh2 at the level of the PET posterior region, and subsequently in the *atrium* and inflow tract (IFT). The scheme illustrates the complementary location in cranial-caudal trend of miR-133a (blue) in comparison with RhoA, Cdc42 and Raldh2 (orange).

As illustrated in Figure 2, by using in situ hybridisation (ISH), we report the expression pattern of miR-133a, from primitive endocardial tubes to the early formation of cardiac *asa*, where the intensity is decreasing in the anterior-posterior sense, disappearing in the inflow tract region. On the other hand, by using immunohistochemistry (IMH), we observe the location of RhoA and Cdc42, at the same stages, being more intense in the inflow tract region and decreasing in the posterior-anterior sense. This pattern coincides with the cardiac distribution of Raldh2.

**Figure 3:**
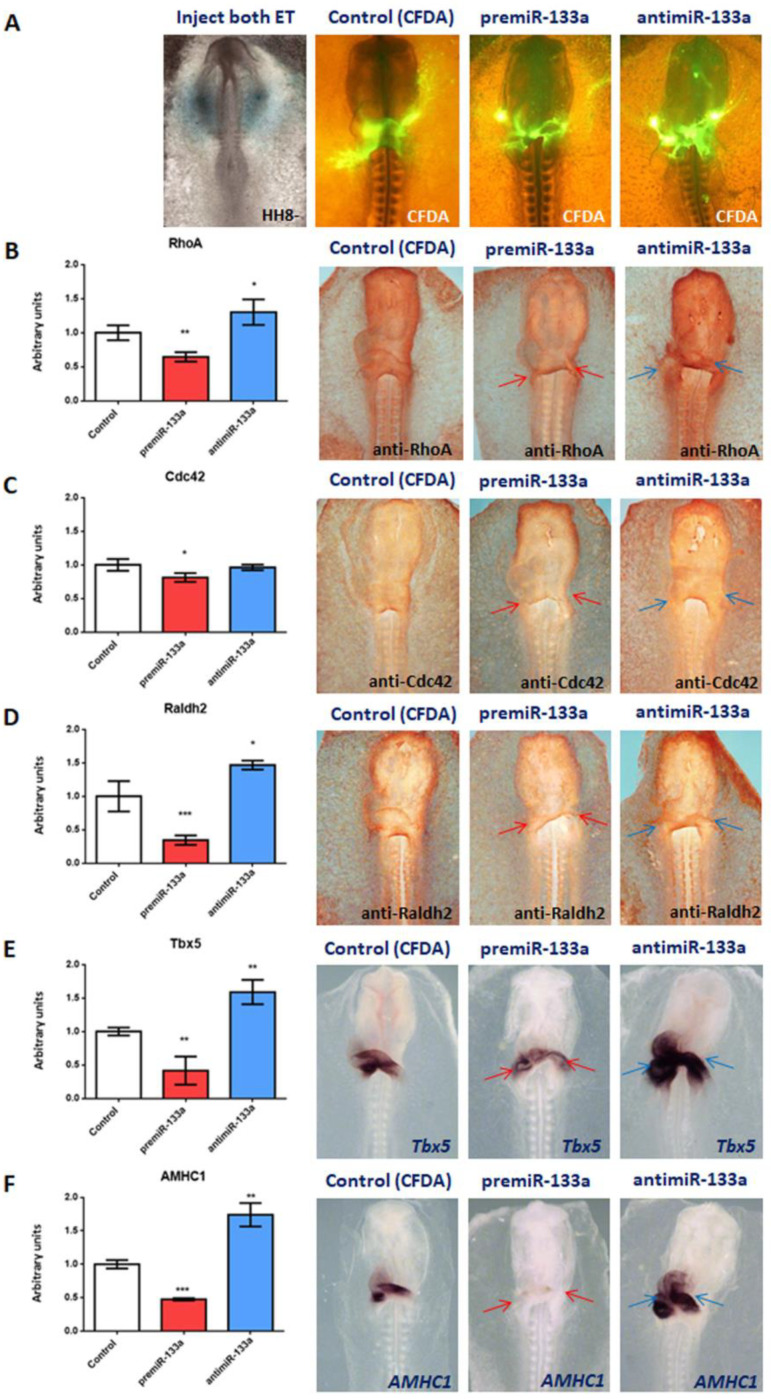
Effect of miR-133a gain- and loss-of-function on posterior cardiac segment. Whole-mount IMH for RhoA, Cdc42 and Raldh2. and ISH for *Tbx5* and *AMHC1.* Embryos microinjected with CFDA (control), premiR-133a or antimiR-133a, at the level of the posterior cardiac precursors of both primitive endocardial tubes, and visualisation of CFDA (**A**). Note that, at the *atrium* and inflow tract levels, RhoA (**B**), Cdc42 (**C**), Raldh2 (**D**), *Tbx5* (**E**) and *AMHC1* (**F**) are dramatically reduced, and an atrophic sino-atrial region in the heart tube after premiR-133a treatment is indicated by the red arrows, whereas they are markedly increased and expanded after miR-133a inhibition (blue arrows). RT-qPCR of RNA from dissected cardiac *asa* (left side) in embryos microinjected either with CFDA, premiR-133a or antimiR-133a. A high level of miR-133a leads to decreased RhoA, Cdc42, Raldh2, *Tbx5* and *AMHC1* transcripts, whereas miR-133a inhibition leads to increased transcripts. The standard deviations are from three independent experiments. Student’s *t*-test: * *p* < 0.05, ** *p* < 0.01, *** *p* < 0.005 with respect to control (CFDA) embryos.

To analyse the functional role of these complementary anterior-posterior patterns, we performed miR-133a gain- and loss-of-functions experiments by injecting premiR-133a and antimiR-133a, respectively, into the posterior cardiac precursors of both primitive endocardial tubes (Figure 3). The control and experimental embryos were analysed with anti-RhoA and anti-Cdc42 antibodies. Furthermore, a group of cardiac *asa* were dissected (Supplementary Figure S2) and subsequently subjected to RNA extraction and qPCR analysis. Our results show that miR-133a represses RNA and protein levels of RhoA and Cdc42 genes in the *atrium* and *sinus venosus* (Figure 3B–C). Supporting these results, our loss-of-functions experiments by means of antimiR-133a demonstrate the opposite effects. In line with our gain- and loss-of-function experiments, we found that miR-133a inhibits RNA and protein levels of the Raldh2 gene, and antimiR-133a induces it (Figure 3D). Since bioinformatics analyses do not show any evidence of Raldh2 as a putative target of miR-133a, our results suggest that Raldh2 would be indirectly modulated by miR-133a, by trigger mRNA degradation of those two pivotal genes, RhoA and Cdc42, required for Raldh2 expression and therefore for the adequate synthesis of RA.

### 2.2. miR-133a Suppresses Tbx5 and AMHC1 Expression

In this study, we also analyse the effect of miR-133a on *Tbx5* and *AMHC1* expressions during cardiac atrial differentiation (Figure 3E,F). Our gain-of-function experiments show significant downregulation of both atrial markers, followed by a reduction in size of the corresponding area located in the posterior segment of the cardiac loop compared to the control embryos. In line with these data, loss-of-function experiments presented increased expression of these markers in most of the cardiac loop region. Additionally, our qPCR analyses supported these findings. Altogether, these data reveal a negative modulation of endogenous *Tbx5* and *AMHC1* expressions by miR-133a during cardiac tube formation.

### 2.3. miR-133a Modulates Cellular Proliferation during Posterior Differentiation of the Cardiac Tube

**Figure 4:**
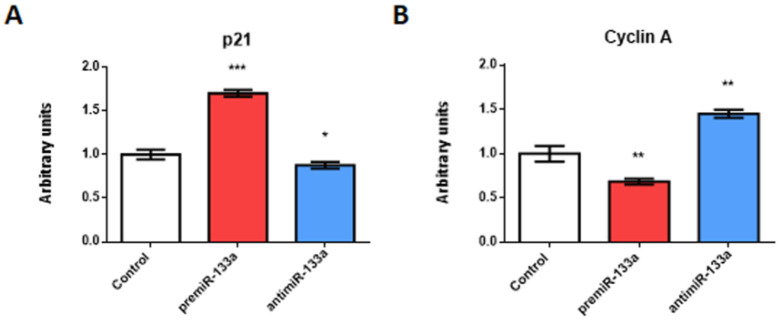
Effect of miR-133a gain- and loss-of-function experiments on cellular proliferation during posterior differentiation of cardiac tube. RT-qPCR of RNA from dissected cardiac *asa* in embryos microinjected either with CFDA, premiR-133a or anti-miR-133a, at the level of the posterior cardiac precursors of both primitive endocardial tubes. Note that miR-133a treatment leads to increased p21 (**A**) and decreased cyclin A (**B**) transcripts, whereas miR-133a inhibition leads to decreased p21 (**A**) and increased cyclin A (**B**) transcripts. The standard deviations are from three independent experiments. Student’s *t*-test: * *p* < 0.05, ** *p* < 0.01, *** *p* < 0.005 with respect to control (CFDA) embryos.

In order to determine the specific role of miR-133a in cellular proliferation during posterior cardiac segment differentiation, in this study, we analyse the expression of two key drivers of cellular proliferation: p21 and cyclin A (Figure 4). A group of cardiac *asa* were dissected from experimental embryos (Supplementary Figure S2) and subsequently subjected to RNA extraction and qPCR analysis. Our gain-of-function experiments show that miR-133a upregulates p21, while represses cyclin A. In accordance with these data, our loss-of-function experiments show downregulation of p21 and upregulation of cyclin A. Since p21 and cyclin A are modulated by RhoA and Cdc42, respectively, our results suggest indirect modulation of miR-133a of those proliferative modulators.

**Figure 5:**
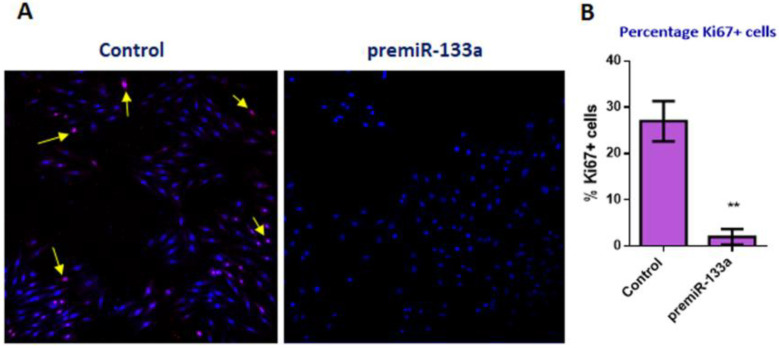
Effect of miR-133a on cellular proliferation in cardiomyocytes. Immunohistochemistry for Ki67 positive cells (arrows) and staining with DAPI (blue) in control cardiomyocytes obtained from H9c2 cell culture and subjected to premiR-133a treatment (**A**). Note that the percentage of Ki67 positive cells is dramatically repressed in premiR-133a treated cardiomyocytes (**B**). Student’s *t*-test: ** *p* < 0.01.

Furthermore, by means of in vitro miR-133a gain-of-function experiments on cardiomyocytes derived from undifferentiated H9c2 cells (Figure 5), we observe that the Ki67 proliferative marker decreased, accompanied by a lower number of cells compared with the control, suggesting that miR-133a exerts as a negative microRNA proliferator.

### 2.4. Retinoic Acid Represses miR-133a Expression

**Figure 6:**
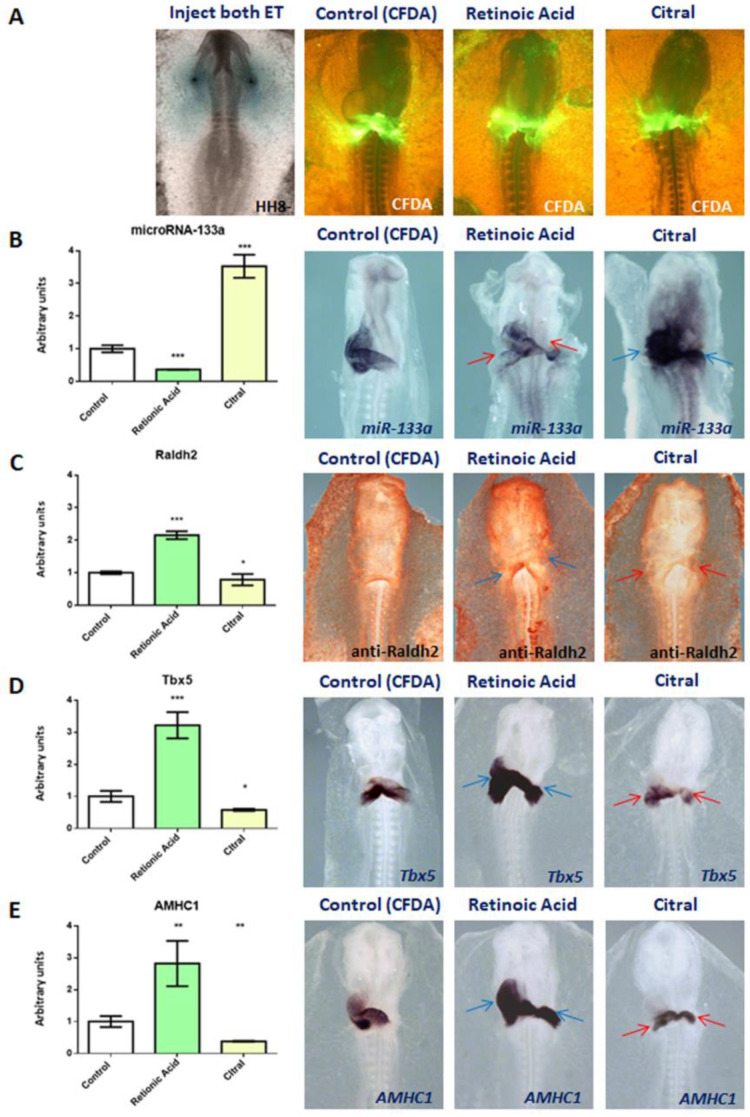
Whole-mount ISH for miR-133a, *Tbx5*, *AMHC1* and IMH for Raldh2. Embryos microinjected with CFDA (control), Retinoic acid (RA) or Citral, at the level of the posterior cardiac precursors into both primitive endocardial tubes, and visualisation of CFDA (**A**). The gain-of-function of RA leads to diminished miR-133a expression (red arrows) at the cardiac *asa* level (**B**), accompanied by increased protein levels of Raldh2 (**C**) and expanded expression of *Tbx5* (**D**) and *AMHC1* (**E**) in the heart tube and in the inflow tract (blue arrows). Note atrophic sino-atrial region with increased miR-133a expression (blue arrows) at cardiac *asa* level (**B**), and also (red arrows) diminished Raldh2 protein level (**C**), and decreased *Tbx5* (**D**) and *AMHC1* (**E**) expressions by RA synthesis inhibition. RT-qPCR of RNA from dissected cardiac *asa* (left side) in embryos microinjected either with CFDA, RA or Citral. The standard deviations are from three independent experiments. Student’s *t*-test: * *p* < 0.05, ** *p* < 0.01, *** *p* < 0.005 with respect to control (CFDA) embryos.

Since our results reveal that miR-133a represses RA synthesis, we analysed the relationship between these two factors (Figure 6). We then performed gain-of-function experiments by means of RA microinjections into both primitive endocardial tubes (Figure 6A), as well as loss-of-function experiments by using Citral injection, described as an inhibitor of RA synthesis [13,49,50]. Our results show that RA administration inhibits miR-133a expression at the cardiac *asa* level. In line with this, increased areas of miR-133a expression are observed after Citral injections. Our qPCR analysis supported the above findings (Figure 6B). Furthermore, RA administration increased RNA and protein levels of the Raldh2 gene, being decreased by Citral administration (Figure 6C). Moreover, we observed that *Tbx5* and *AMHC1* expressions in the cardiac loop stage were increased after RA injection as compared to control embryos. In this line, there was an inhibition of these atrial gene markers after Citral administration, also supported by qPCR analysis (Figure 6D,E). All the above results suggest a narrow interaction between RA and miR-133a during differentiation and early development of the posterior cardiac tube segment.

## 3. Discussion

In the last few years, microRNAs have been proven to play relevant functions during embryogenesis, including cardiovascular development. In previous studies, we have shown that miR-133a, in particular, is involved in the early stages of cardiogenesis [44]. We also observed that RA signalling participates in atrial chamber differentiation during early cardiac looping [13]. Furthermore, it has been reported that RA is necessary to define the anterior-posterior boundaries of the heart-forming mesoderm (even before looping) and to form the *atrium* and *sinus venosus* [16]. It is well known that Raldh2, with an expression pattern mostly restricted to the caudal region and displaying a decreasing caudal-cranial trend, is largely involved in RA synthesis, which signal is essential to promote atrial cell identity within the cardiac progenitor fields [19,20,21,22,23,24].

**Figure 7:**
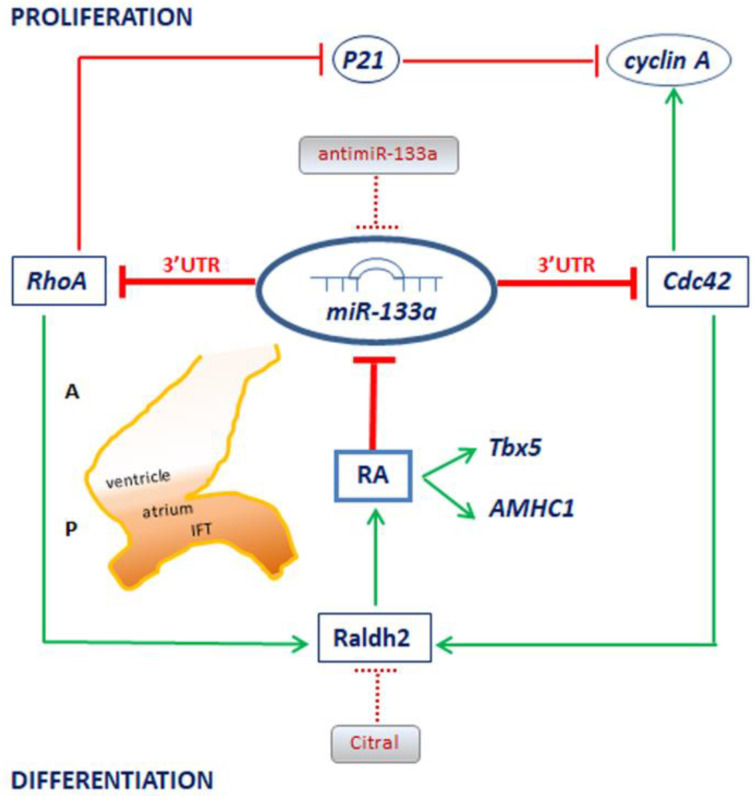
Model proposed for the interplay between miR-133a and RA during posterior heart tube formation. Our model indicates that miR-133a downregulates RhoA and Cdc42. Consequently, Raldh2 downregulation is induced by miR-133a, via these Rho GTPases. RA synthesis is Raldh2-dependent. Thus, miR-133a modulates RA signalling via Raldh2 expression. Also, RA negatively modulates miR-133a expression during the early genetic programme of the sinoatrial region. Additionally, our model indicates that miR-133a modulates cell proliferation by acting on the cell cycle regulators p21 (via RhoA) and cyclin A (via Cdc42). We hypothesise that there is a negative feedback mechanism between miR-133a and RA signalling during early development of the posterior cardiac tube segment. A: anterior segment, P: posterior segment.

In this study, we carried out a detailed analysis in order to establish a relationship between miR-133a and RA during posterior cardiac tube segment differentiation. Our results show that miR-133a downregulates Raldh2 expression. Nevertheless, we did not detect that miR-133a recognises Raldh2 3′UTR. Therefore, we explored the possible interaction between miR-133a and Rho GTPases, RhoA and Cdc42, described as relevant factors linked to Raldh2 expression [32]. Our findings show that miR-133a recognises RhoA and Cdc42 3′UTRs, interactions that are translated into repression of mRNA and codified protein levels of both genes, which have been observed in humans and mice [46]. Moreover, we observed that miR-133a downregulates RhoA and Cdc42. Consequently, it is evident that Raldh2 downregulation is induced by miR-133a via these Rho GTPases. Additionally, we observed miR-133a expression pattern mostly located in the cranial region, showing a decreasing cranial-caudal trend, while RhoA and Cdc42 are located—similar to Raldh2—in the caudal region, decreasing cranially. Taking into account all of the above, our proposed model illustrates that miR-133a modulates RA signalling via Raldh2 expression, a fundamental mechanism to differentiate the posterior cardiac tube segment (Figure 7).

On the other hand, our results show that RA administration downregulates miR-133a expression. In fact, RA modulates many microRNA expressions to exert their biological functions, establishing a large number of regulatory networks currently under study [48]. Furthermore, we observed in this work that RA administration upregulates atrial genes *Tbx5* and *AMHC1,* as we reported in previous studies [13], in agreement with other authors [25,28,51,52]. As a novelty, our experiments reveal that miR-133a downregulates both atrial gene expressions. In addition, our findings obtained by means of Citral administration indicate that the blockage of endogenous RA synthesis gives rise to Raldh2 inhibition, in parallel to that of *Tbx5* and *AMHC1.* In accordance with previous works [26,53], in our study we established a correlation between Raldh2, an RA synthetic enzyme, and *Tbx5* and *AMHC1,* both RA inducible genes, coincident with *Tbx5* and *AMHC1* upregulation cranially after RA injections in the prospective atrial cells. Consequently, in our experiments, exogenous RA leads to atrialisation of the heart, whereas inhibition of RA synthesis causes an ablated atrial chamber, in line with previous authors [25]. Significantly, our miR-133a gain-of-function experiments led to a reduction in the size of the corresponding sinoatrial domain together with lower expression of *Tbx5* and *AMHC1*. Therefore, we hypothesise that, through this network, miR-133a modulates the posterior cardiac tube differentiation. Thus, our model (see Figure 7) supports the fact that there is a negative feedback mechanism between miR-133a and RA signalling during *sino-atrium* formation.

It is well established that growth and differentiation processes require a modulatory cardiomyocyte proliferation factor [41,46,54]. In the absence of miR-133a expression there is an ectopic expression of smooth muscle genes in the heart, as well as aberrant cardiomyocyte proliferation. These abnormalities have been attributed, at least in part, to elevated expression of serum response factor (SRF) and cyclin D2, which are targets of miR-133a. SRF regulates the genes responsible for cardiac muscle and smooth muscle differentiation and growth, and cyclin D2 promotes cardiomyocyte cell cycle progression [41]. Moreover, it has been proposed in humans and mice, that miR-133a plays a crucial role in determining cardiomyocyte hypertrophy by means of RhoA and Cdc42 modulation [46]. RhoA has been implicated in the regulation of hypertrophic cardiac muscle cell growth. Transgene-positive mice expressing high levels of activated RhoA showed pronounced atrial enlargement and manifested a lethal phenotype [55]. Cdc42 functions as a cell cycle progression determinant [56]. Thus, inactivation of Cdc42 causes embryonic lethality with heart development defects [57]. Increased cardiac expression of Rho GDI reduces the proliferation of cardiomyocytes. The mechanisms affecting cell proliferation in transgenic hearts include upregulation of p21, consistent with inhibition of RhoA, and downregulation of cyclin A, consistent with RhoA and Cdc42 inhibition by Rho GDI [32]. In line with this, our cardiac tube experiments revealed that miR-133a modulates the cell cycle regulators p21 and cyclin A. Additionally, our H9c2 culture cells experiments showed that miR-133a diminishes proliferative marker Ki67.

Considering all the above data, we hypothesise that miR-133a modulates differentiation and proliferation during posterior heart tube formation (Figure 7). In this line of research, several reports have pointed out the relevant roles of miR-133a in heart development and cardiac diseases, considering it to be a basis for adult heart regeneration and repair [58,59,60,61]. Noticeably, miR-133a has been described as a protective factor in myocardial infarction by regulating cardiac remodelling [62]. In addition, low circulating levels of RA have recently been shown to reduce the expression of critical RA-dependent gene programmes in heart failure [63]. Taking all these data into account, the relationship between miR-133a and RA described in our model may provide new insights into cardiac development and heart diseases as biomarkers and/or therapy.

## 4. Materials and Methods

Experimental protocols with animals were performed in agreement with the Spanish law in application of the EU Guidelines for animal research and conformed to the Guide for the Care and Use of Laboratory Animals, published by the US National Institutes of Health (NIH Publication No.85–23). Approval from the University of Extremadura bioethics board was obtained prior to the initiation of the study.

### 4.1. Early Chick Whole Embryo Culture

Fertilised eggs (Granja Santa Isabel, Córdoba, Spain) were incubated at 38 °C in forced draft humidified incubators. Embryos were staged [64,65,66] and subjected to early chick (EC) embryo culture [67].

### 4.2. Embryo Injections into the Posterior Cardiac Precursors of Both Primitive Endocardial Tubes

Stage HH 7–8 cultured embryos were microinjected (using an Inject + Matic microinjector system) in both primitive endocardial tubes into the posterior cardiac precursors committed to sino-atrial fates [23,24]. For gain-of-function experiments, two different groups of embryos were microinjected, with premiR-133a and all-trans retinoic acid (RA), respectively. Likewise, for loss-of-function experiments, two different groups of embryos were microinjected, with antimiR-133a and an inhibitor of retinoic acid synthesis, Citral (3,7-dimethyl-2,6-octadienal), respectively. For control embryos, CFDA (carboxyfluoresceindiacetate, succinidyl ester) was microinjected.

For microRNAs premiR-133a (Ambicon) or antimiR-133a (Ambion), a working solution that contained a final concentration of 1 μM, 2.5 mM CFDA (Molecular Probes) and 1/10 volume of 0.5% (wt/vol) fast green FCF, was prepared.

A working solution that contained a final concentration of 10 µg/mL (RA) or 10 mmol/L (Citral), 2.5 mM CFDA (Molecular Probes) and 1/10 volume of 0.5% (wt/vol) fast green FCF was prepared.

After 16–18 h of additional incubation, embryos were photographed under bright and fluorescent light (Nikon digital, SIGHT DS-U1) and were selected according to the location and extent of the injection. The selected embryos were either fixed in 4% PFA, processed for gene expression (ISH) or immunochemistry (IMH) analysis, or cardiac loops were collected for RNA isolation.

### 4.3. Whole-Mount In Situ Hybridisation (ISH)

Two different ISH-procedures were performed following our previous procedure [68]. One group of Control, RA, Citral and control (CFDA) embryos was processed [69] for LNA-ISH using miR-133a LNA-labelled microRNA probe (miRCURY LNA™ Detection probe 5′-DIG and 3′-DIG labelled, Exiqon). Other groups of experimental and control (CFDA) embryos were processed for ISH following standard procedure [70] using antisense-*Tbx5* and -*AMHC1* labelled probes [13].

### 4.4. Whole-Mount Immunohistochemistry (IMH)

Experimental and control (CFDA) embryos were subjected to whole mount IMH was performed as previously described by us [68,71], using RhoA Polyclonal Antibody Rabbit (1:300, Invitrogen, OSR00266W), ALDH1A2 Polyclonal Antibody Rabbit (1:100, Invitrogen PA5-22377), followed by Goat anti-rabbit IgG-HRP antibody (1:1000, Upstate, 12-348) and Cdc42 monoclonal antibody Mouse (1:50, Santa-Cruz, sc-8401), followed by a Goat anti-mouse IgG-HRP antibody (1:200, Jackson Inmuno Research 115-035-003).

### 4.5. RNA Isolation and qRT-PCR

Cardiac loops isolated from experimental and control (CFDA) embryos (Supplementary Figure S2) were subjected to qRT-PCR analysis following MIQE guidelines [72,73,74]. RNA was extracted and purified using the ReliaPrep RNA Cell Miniprep System Kit (Promega) according to the manufacturer’s instructions. For mRNA expression measurements, 1 μg of total RNA was used for retro-transcription with a Maxima First Strand cDNA Synthesis Kit for qRT-PCR (Thermo Scientific). Real-time PCR experiments were performed with 2 μL cDNA, Go Taq qPCR Master Mix (Promega) and corresponding primer sets (Supplementary Table S1). For microRNA expression analyses, 20 ng of total RNA was used for retro-transcription with Universal cDNA Synthesis Kit II (Exiqon) and the resulting cDNA was diluted 1/80. Real-time PCR experiments were performed with 1 μL of diluted cDNA, Go Taq qPCR Master Mix (Promega) as well. All qPCRs were performed using a CFX384TM thermocycler (Bio-Rad) following the manufacture’s recommendations. The relative expression of each gene was calculated using Gusb and Gadph as internal controls for mRNA expression analyses and 5S and 6U for microRNA expression analyses, respectively [75]. Each PCR reaction was carried out in triplicate and repeated in at least three distinct biological samples to obtain representative means.

### 4.6. Analysis In Silico

Target-Scan software was used to perform bioinformatics analysis of binding site by miR-133a at 3′UTRs of predicted targets as described before [76].

### 4.7. Luciferase Assays and 3T3 Transfection

RhoA and Cdc42 3′UTR constructs (Supplementary Table S2) were PCR amplified and cloned into the pMIR_REPORT vector. 3t3 fibroblasts (ATCC) were co-transfected with 100 ng of different constructs luciferase vector and 300 ng of pcLux vector control for internal normalisation. Luciferase activity (Pierce™ Gaussia Luciferase Flash Assay Kit) was normalised to the pcLux vector control (Pierce™ Cypridina Luciferase Flash Assay Kit) and compared to non-transfected controls. Each luciferase assay was carried out in triplicate and repeated in at least three distinct biological samples to obtain representative assays.

### 4.8. H9c2 Culture Cells and Transfection

The H9c2 cell line (kindly provided by Dr. Paulo J. Oliveira, Coimbra, Portugal) was cultured in DMEM medium supplemented with 10% foetal bovine serum, 100 U/mL penicillin and 100 μg/mL streptomycin in 100 cm^2^ culture disks at 37 °C in a humidified atmosphere of 5% CO_2_. Cells were fed every 2–3 days. H9c2 cells (6 × 10^5^ cells per well) were transfected with premiR-133a (Thermofisher) as previously described [77].

### 4.9. Immunohistochemistry in H9c2 Cell Cultures

Control and experimentally premiR-133a treated cells were rinsed in PBS for 10 min at room temperature and fixed with 1% PFA for 2 h at 4 °C. After fixation, the samples were rinsed three times (10 min each) in PBS at room temperature and then permeabilysed with 1% Triton X-100 in PBS for 30 min at room temperature. To block nonspecific binding sites, PBS containing 5% goat serum and 1% bovine serum albumin (Sigma, St. Louis, MO, USA) was applied to the cell cultures overnight at 4 °C. Subsequently, they were incubated with DAPI (1:1000; Sigma) for 7 min at room temperature and rinsed three times in PBS for 5 min each. Alternatively, for DAPI staining, control and experimentally premiR-133a treated cells were immunofluorescently labelled to detect Ki67 expression. Primary antibodies against Ki67 (ab16667) were used, diluted (1:200) in PBS, and applied to each culture overnight at 4 °C. Subsequently, the samples were rinsed three times (for 1 h each) in PBS to remove excess primary antibody and incubated 2 h at room temperature with Alexa-Fluor 546 anti-rabbit (1:100; Invitrogen) as the secondary antibody. Cell cultures were stored in PBS in darkness at 4 °C until analysed using a Leica TCS SP5 II confocal scanning laser microscope.

### 4.10. Statistical Analyses

For statistical analyses of datasets, unpaired Student’s *t*-tests were used, as previously reported [73,78]. Significance levels or *p*-values are stated in each corresponding figure legend. A *p*-value < 0.05 was considered statistically significant.

## Data Availability

Not applicable.

## Supplementary Materials

The following supporting information can be downloaded at: https://www.mdpi.com/article/10.3390/ijms23084179/s1.
