# Supplementary material for: Inhibition of RhoA and Cdc42 by miR-133a Modulates Retinoic Acid Signalling during Early Development of Posterior Cardiac Tube Segment
Source: Int J Mol Sci. 2022 Apr 10;23(8):4179. doi: 10.3390/ijms23084179 (PMC9025022; doi:10.3390/ijms23084179)
Supplement: Supplementary file 1 [file ijms-23-04179-s001.zip › ijms-1656258-supplementary.pdf]

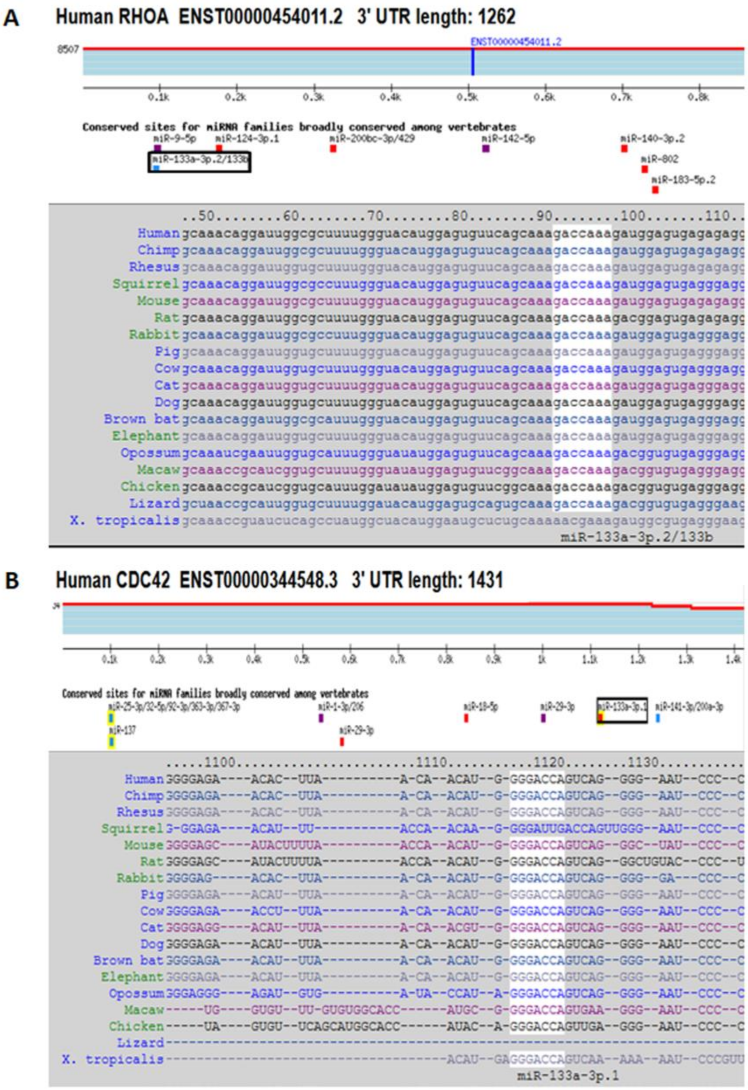

Supplementary Figure S1. TargetScan analysis of putative microRNA binding sites in Human RhoA 3´UTR and Cdc42 3´UTR. As can be observed, miR-133a is a putative microRNA targeting Rho A (panel A), and Cdc42 (panel B). Note their binding site are highly conserved along the RhoA and Cdc42 3´UTR in several species, including chicken.

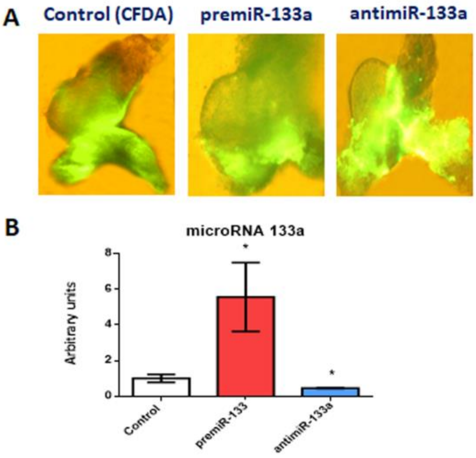

Supplementary Figure S2. Panel **A** shows the visualization of CFDA in dissected cardiac *asa* (HH11) from embryos microinjected, either with CFDA, premiR-133a or antimiR-133a, at the level of the posterior cardiac precursors into both primitive endocardial tubes. Panel **B** shows qRT-PCR results validating the miR-133a gain- and loss-of-function respect to control on cardiac *asa*. Student's t-test: *$p < 0.05$ with respect to control (CFDA) embryos.

qPCR primers		
Gene	Specie	Sequence
Tbx5_Forward	Gallus Gallus	AACCCCTACCCGATCTCCCC
Tbx5_Reverse	Gallus Gallus	CCATGTAGGGCTTCTTGTAGGG
AMHC1_Forward	Gallus Gallus	AAGACCGGAAGAACATGGTG
AMHC1_Reverse	Gallus Gallus	GAGACTCAGCCATGTCAGCA
RhoA_Forward	Gallus Gallus	ATTTCTGTCCCAACGTGCCT
RhoA_Reverse	Gallus Gallus	CAAAAACCTCCCTCACACCG
Cdc42_Forward	Gallus Gallus	GCTGTAACAGTGATGATTGGAGG
Cdc42_Reverse	Gallus Gallus	TCTGTGGATAGCTGAGGGGT
Raldh2_Forward	Gallus Gallus	ACTGAGATTTAAAACGGTGGATGA
Raldh2_Reverse	Gallus Gallus	TCTCTCTACCATTGCCAGACA
p21_Forward	Gallus Gallus	AGCCAACACAACAGAAGAGGA
p21_Reverse	Gallus Gallus	TCCCATGGAGACCGACGATA
Cyclin A_Forward	Gallus Gallus	TAAGCCTTGCCTCATGGACC
Cyclin A_Reverse	Gallus Gallus	TCTGGTGCGTCAATAAGCGA
Gapdh_Forward	Gallus Gallus	TGTCCTCTCTGGCAAAGTCC
Gapdh_Reverse	Gallus Gallus	TGCCCATTGATCACAAGTTT

Supplementary Table S1. Primer sequences and corresponding genes for qRT-PCR (Gallus gallus).

3’UTRs cloning primers		
RhoA_3’UTR_F	Gallus Gallus	GGACTAGTGAGCCTGTCAAACCTGAGG
RhoA_3’UTR_R	Gallus Gallus	GGAAGCTTTGCTTGGCCATCTTCCTCT
Cdc42_3’UTR_F	Gallus Gallus	GGGAAGCTTATTGGAATGAGAGCTGGCCC
Cdc42_3’UTR_R	Gallus Gallus	GGGACTAGTATCCTTGTTGGGCTGGAACC

Supplementary Table S2.
Primer sequences 3’UTR corresponding RhoA and Cdc42 genes for luciferase assays (Gallus gallus).
